# Supplementary figures and images for: Genetic diversity of the two-spotted stink bug Bathycoelia distincta (Pentatomidae) associated with macadamia orchards in South Africa
Source: PLoS One. 2022 Jun 10;17(6):e0269373. doi: 10.1371/journal.pone.0269373 (PMC9187107; doi:10.1371/journal.pone.0269373)

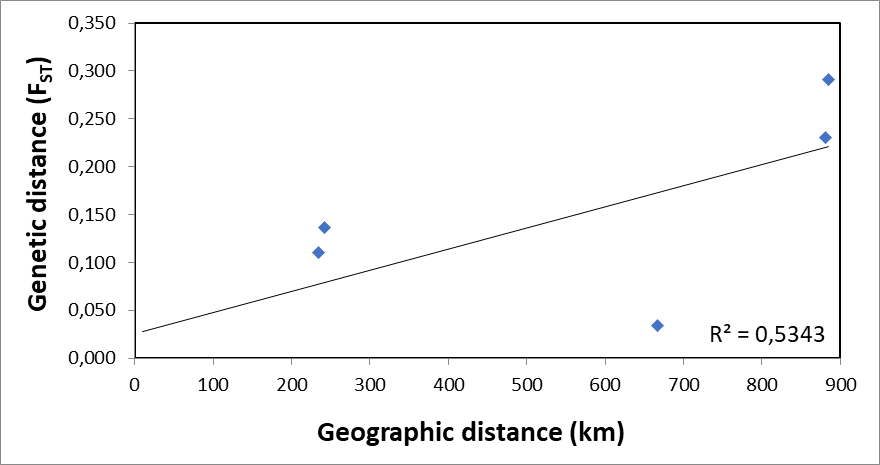

Supplement: S1 Fig — (TIF) [file pone.0269373.s001.tif]

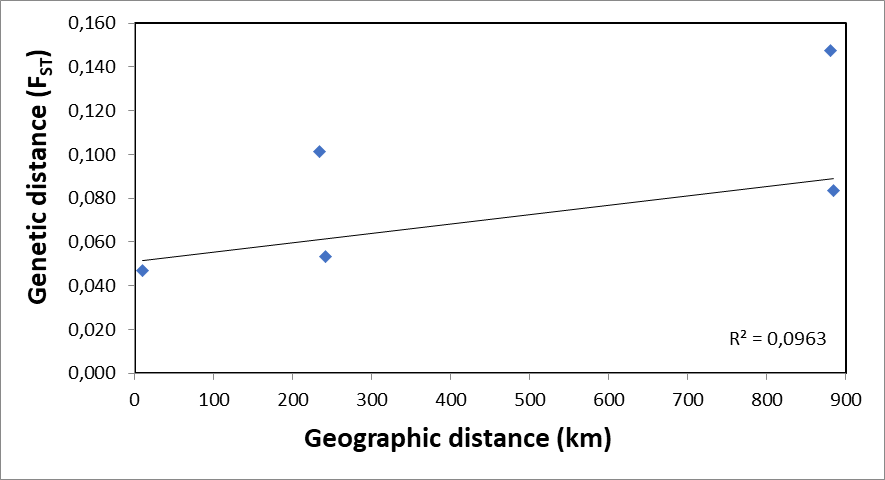

Supplement: S2 Fig — (TIF) [file pone.0269373.s002.tif]

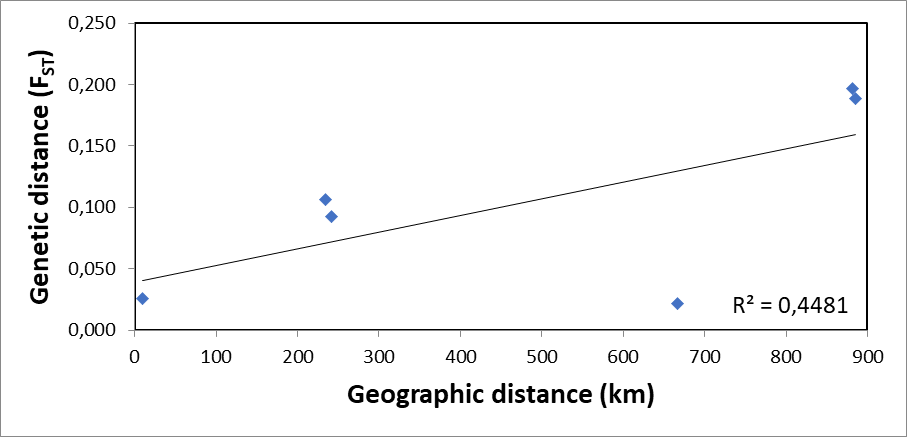

Supplement: S3 Fig — (TIF) [file pone.0269373.s003.tif]
