# Supplementary material for: Genetic diversity of the two-spotted stink bug Bathycoelia distincta (Pentatomidae) associated with macadamia orchards in South Africa
Source: PLoS One. 2022 Jun 10;17(6):e0269373. doi: 10.1371/journal.pone.0269373 (PMC9187107; doi:10.1371/journal.pone.0269373)
Supplement: S1 Table — (DOCX) [file pone.0269373.s004.docx]

| Haplotype | n | Individuals |
| --- | --- | --- |
| Hap_C1 | 1 | MSL1 |
| Hap_C2 | 63 | MSL2; MSL3; MSL6; MSL7; MSL9; MSL11; MSL12; MSL13; MSL14; MSL15; MSL16; MSL19; MSL20; MSL21; MSL22; MSL23; MSL25; MSL26; MSL27; MSL29; MSL31; MSL36; MSL37; MSL38; MSL42; MSL43; MSL45; MSL47; MSL49; MSL50; MSL52; MSL54; MSL55; MSL56; MSL61; MSL62; MSL63; MSL66; MSM5; MSM9; MSM11; MSM19; MSM21; MSM23; MSM30; MSM36; MSM39; MSM40; MSM41; MSM45; MSK8; MSK10; MSK21; MSK23; MSK26 ; MSK36 ; MSK38; MSK42; MSK43; MSK44; MSK45; MSK46; MSM28 |
| Hap_C3 | 1 | MSL4 |
| Hap_C4 | 3 | MSL17; MSM10; MSM27 |
| Hap_C5 | 1 | MSL32 |
| Hap_C6 | 1 | MSL35 |
| Hap_C7 | 1 | MSL39 |
| Hap_C8 | 1 | MSL46 |
| Hap_C9 | 1 | MSL51 |
| Hap_C10 | 1 | MSM1 |
| Hap_C11 | 1 | MSM20 |
| Hap_C12 | 1 | MSM22 |
| Hap_C13 | 2 | MSM29; MSL60 |
| Hap_C14 | 1 | MSK27 |
| Hap_C15 | 1 | MSL10 |
| Hap_C16 | 1 | MSL34 |
| Hap_C17 | 3 | MSL24; MSL30; MSM38 |
| Hap_C18 | 1 | MSL41 |
| Hap_C19 | 50 | MSL18; MSL65; MSM2; MSM3; MSM4; MSM7; MSM8; MSM12; MSM13; MSM14; MSM15; MSM16; MSM17; MSM18; MSM24; MSM25; MSM31; MSM32; MSM34; MSM35; MSM37; MSM44; MSK1; MSK2; MSK3; MSK4; MSK5; MSK6; MSK7; MSK9; MSK11; MSK14; MSK15; MSK16; MSK17; MSK19; MSK20; MSK22; MSK25; MSK28; MSK29; MSK30; MSK31; MSK32; MSK33; MSK34; MSK35; MSK37; MSK39; MSK40 |
| Hap_C20 | 1 | MSK13 |
| Hap_C21 | 1 | MSK13 |
| Hap_C22 | 3 | MSM6; MSM26; MSM33 |
| Hap_C23 | 1 | MSM42 |
| Hap_C24 | 1 | MSL28; MSL44; MSL57 |
| Hap_C25 | 1 | MSL59 |
| Hap_C26 | 1 | MSK41 |
| Hap_C27 | 1 | MSK12 |
| Hap_C28 | 1 | MSL64 |
| Hap_C29 | 1 | MSK24 |
| Hap_C30 | 1 | MSL48 |
| Hap_C31 | 1 | MSL5 |
| Hap_C32 | 3 | MSL33; MSL53; MSL58 |
| Hap_C33 | 1 | MSL8 |
| Hap_C34 | 1 | MSM43 |
| Hap_C35 | 1 | MSL40 |
