# Supplementary material for: Genetic diversity of the two-spotted stink bug Bathycoelia distincta (Pentatomidae) associated with macadamia orchards in South Africa
Source: PLoS One. 2022 Jun 10;17(6):e0269373. doi: 10.1371/journal.pone.0269373 (PMC9187107; doi:10.1371/journal.pone.0269373)
Supplement: S2 Table — (DOCX) [file pone.0269373.s005.docx]

| Haplotype | n | Individuals |
| --- | --- | --- |
| Hap_B1 | 105 | MSL39; MSL41; MSL43; MSL45; MSL47; MSL48; MSL53; MSL55; MSL58; MSL60; MSL61; MSL62; MSL64; MSL65; MSL14; MSL18; MSL17; MSL20; MSL22; MSL25; MSL33; MSL34; MSL35; MSL36; MSL38; MSL1; MSL3; MSL4; MSL5; MSL7; MSL10; MSK17; MSM18; MSM21; MSM22; MSM23; MSM24; MSM25; MSM27; MSM29; MSM30; MSM31; MSM32; MSM33; MSM37; MSM38; MSM39; MSM42; MSM43; MSM44; MSM45; MSM1; MSM2; MSM3; MSM4; MSM5; MSM6; MSM7; MSM8; MSM9; MSM11; MSM12; MSM13; MSM14; MSM15; MSK15; MSK16; MSK17; MSK18; MSK19; MSK20; MSK21; MSK22; MSK23; MSK24; MSK25; MSK26; MSK27; MSK28; MSK29; MSK30; MSK31; MSK32; MSK33; MSK34; MSK35; MSK37; MSK38; MSK39; MSK42; MSK43; MSK44; MSK45; MSK46; MSK10; MSK11; MSK13; MSK6; MSK7; MSK9; MSK1; MSK2; MSK3; MSK4; MSK5 |
| Hap_B2 | 10 | MSL46; MSL52; MSL11; MSL13; MSL19; MSL37; MSL9; MSM36; MSM41; MSK14 |
| Hap_B3 | 1 | MSL49 |
| Hap_B4 | 1 | MSL56 |
| Hap_B5 | 2 | MSL63; MSL6 |
| Hap_B6 | 1 | MSL12 |
| Hap_B7 | 1 | MSL31 |
| Hap_B8 | 1 | MSL32 |
| Hap_B9 | 1 | MSL2 |
| Hap_B10 | 1 | MSM10 |
| Hap_B11 | 1 | MSK41 |
| Hap_B12 | 1 | MSK36 |
| Hap_B13 | 1 | MSK40 |
| Hap_B14 | 1 | MSK8 |
| Hap_B15 | 1 | MSL21 |
| Hap_B16 | 1 | MSM40 |
| Hap_B17 | 2 | MSL42; MSL66 |
| Hap_B18 | 5 | MSL44; MSL51; MSL54; MSL15; MSL23 |
| Hap_B19 | 1 | MSL50 |
| Hap_B20 | 1 | MSL57 |
| Hap_B21 | 1 | MSL59 |
| Hap_B22 | 1 | MSL8 |
| Hap_B23 | 2 | MSM19; MSM28 |
| Hap_B24 | 1 | MSL30 |
| Hap_B25 | 1 | MSK12 |
| Hap_B26 | 1 | MSM16 |
| Hap_B27 | 1 | MSL40 |
| Hap_B28 | 1 | MSM20 |
| Hap_B29 | 1 | MSM26 |
| Hap_B30 | 1 | MSM34 |
| Hap_B31 | 1 | MSM35 |
| Hap_B32 | 4 | MSL26; MSL27; MSL29; MSL16 |
| Hap_B33 | 1 | MSL28 |
| Hap_B34 | 1 | MSL24 |
