# Supplementary material for: Genetic diversity of the two-spotted stink bug Bathycoelia distincta (Pentatomidae) associated with macadamia orchards in South Africa
Source: PLoS One. 2022 Jun 10;17(6):e0269373. doi: 10.1371/journal.pone.0269373 (PMC9187107; doi:10.1371/journal.pone.0269373)
Supplement: S3 Table — (DOCX) [file pone.0269373.s006.docx]

| Haplotype | n | Individuals |
| --- | --- | --- |
| Hap_CB1 | 1 | MSL1 |
| Hap_CB2 | 1 | MSL2 |
| Hap_CB3 | 32 | MSL3; MSL7; MSL14; MSL20; MSL22; MSL25; MSL36; MSL38; MSL43; MSL45; MSL47; MSL55; MSL61; MSL62; MSM5; MSM9; MSM11; MSM21; MSM23; MSM30; MSM39; MSM45; MSK10; MSK21; MSK23; MSK26; MSK38; MSK42; MSK43; MSK44; MSK45; MSK46 |
| Hap_CB4 | 1 | MSL4 |
| Hap_CB5 | 1 | MSL5 |
| Hap_CB6 | 2 | MSL6; MSL63 |
| Hap_CB7 | 1 | MSL8 |
| Hap_CB8 | 8 | MSL9; MSL11; MSL13; MSL19; MSL37; MSL52; MSM36; MSM41 |
| Hap_CB9 | 1 | MSL10 |
| Hap_CB10 | 1 | MSL12 |
| Hap_CB11 | 3 | MSL15; MSL23; MSL54 |
| Hap_CB12 | 4 | MSL16; MSL26; MSL27; MSL29 |
| Hap_CB13 | 45 | MSL18; MSL65; MSM2; MSM3; MSM4; MSM7; MSM8; MSM12; MSM13; MSM14; MSM15; MSM17; MSM18; MSM24; MSM25; MSM31; MSM32; MSM37; MSM44; MSK1; MSK2; MSK3; MSK4; MSK5; MSK6; MSK7; MSK9; MSK11; MSK15; MSK16; MSK17; MSK19; MSK20; MSK22; MSK25; MSK28; MSK29; MSK30; MSK31; MSK32; MSK33; MSK34; MSK35; MSK37; MSK39 |
| Hap_CB14 | 2 | MSL17 MSM27 |
| Hap_CB15 | 1 | MSL21 |
| Hap_CB16 | 1 | MSL24 |
| Hap_CB17 | 1 | MSL28 |
| Hap_CB18 | 1 | MSL30 |
| Hap_CB19 | 1 | MSL31 |
| Hap_CB20 | 1 | MSL32 |
| Hap_CB21 | 3 | MSL33; MSL53; MSL58 |
| Hap_CB22 | 1 | MSL34 |
| Hap_CB23 | 1 | MSL35 |
| Hap_CB24 | 1 | MSL39 |
| Hap_CB25 | 1 | MSL40 |
| Hap_CB26 | 1 | MSL41 |
| Hap_CB27 | 2 | MSL42; MSL66 |
| Hap_CB28 | 1 | MSL44 |
| Hap_CB29 | 1 | MSL46 |
| Hap_CB30 | 1 | MSL48 |
| Hap_CB31 | 1 | MSL49 |
| Hap_CB32 | 1 | MSL50 |
| Hap_CB33 | 1 | MSL51 |
| Hap_CB34 | 1 | MSL56 |
| Hap_CB35 | 1 | MSL57 |
| Hap_CB36 | 1 | MSL59 |
| Hap_CB37 | 2 | MSL60; MSM29 |
| Hap_CB38 | 1 | MSL64 |
| Hap_CB39 | 1 | MSM1 |
| Hap_CB40 | 2 | MSM6; MSM33 |
| Hap_CB41 | 1 | MSM10 |
| Hap_CB42 | 1 | MSM16 |
| Hap_CB43 | 2 | MSM19; MSM28 |
| Hap_CB44 | 1 | MSM20 |
| Hap_CB45 | 1 | MSM22 |
| Hap_CB46 | 1 | MSM26 |
| Hap_CB47 | 1 | MSM34 |
| Hap_CB48 | 1 | MSM35 |
| Hap_CB49 | 1 | MSM38 |
| Hap_CB50 | 1 | MSM40 |
| Hap_CB51 | 1 | MSM42 |
| Hap_CB52 | 1 | MSM43 |
| Hap_CB53 | 1 | MSM43 |
| Hap_CB54 | 1 | MSK12 |
| Hap_CB55 | 1 | MSK13 |
| Hap_CB56 | 1 | MSK14 |
| Hap_CB57 | 1 | MSK18 |
| Hap_CB58 | 1 | MSK24 |
| Hap_CB59 | 1 | MSK27 |
| Hap_CB60 | 1 | MSK41 |
| Hap_CB61 | 1 | MSK36 |
| Hap_CB62 | 1 | MSK40 |
