# Supplementary material for: Genetic diversity of the two-spotted stink bug Bathycoelia distincta (Pentatomidae) associated with macadamia orchards in South Africa
Source: PLoS One. 2022 Jun 10;17(6):e0269373. doi: 10.1371/journal.pone.0269373 (PMC9187107; doi:10.1371/journal.pone.0269373)
Supplement: Attachment — Submitted filename: Response to Reviewers.pdf [file pone.0269373.s008.pdf]

UNIVERSITEIT VAN PRETORIA
UNIVERSITY OF PRETORIA
YUNIBESITHI YA PRETORIA
Faculty of Natural & Agricultural Sciences
Department of Genetics

Dr Patrizia Falabella

Academic Editor

PLOS ONE

Submission of Revised Manuscript

Thank you for your positive feedback regarding our manuscript, entitled “Genetic diversity of the two-spotted stink bug *Bathycoelia distincta* (Pentatomidae) associated with macadamia orchards in South Africa”. We believe that the suggestions made by the reviewers have improved the scientific quality and clarity of our manuscript.

In this revision, we incorporated all the suggestions made by the reviewers, which has resulted in a number of minor changes to our manuscript.

Please find attached our revised manuscript. Our response to the comments and suggestions made by the reviewers can be found in the document below titled “Response to Reviewers”, a marked copy of the manuscript can be found in the document titled “Revised Manuscript with Track Changes” and the unmarked version of the manuscript can be found in the document titled “Manuscript”.

We thank you in advance for your assistance in handling this manuscript further.

Kindest regards,

Dr Gerda Fourie

Corresponding author: gerda.fourie@up.ac.za

Journal requirements

Please note that the page and line numbers referred to in our responses represent those found in the newly submitted manuscript. Furthermore, specific changes are indicated in italics, where appropriate.

1) **Please ensure that your manuscript meets PLOS ONE's style requirements, including those for file naming.**
   - The manuscript and file names of the manuscript was reviewed in order to ensure that all the PLOS ONE's style requirements are met.

2) **In your methods section, please provide additional information regarding the permits you obtained for the work.**
   - No permit is needed in South Africa. An additional section has been added Lines 130-132: "*Ethics statement. No endangered or protected species were involved in this study. No national permissions were required for this study. All work on this project was conducted with permission from landowners".*

3) **Funding statement modifications.**
   - The funding statement was updated online and in the cover letter.

4) **We note that the grant information you provide in the "funding information" and "Financial Disclosure" sections do not match.**

- As mentioned above, funding information was updated and corrected.

5) **We note that Figure 1 in your submission contain map images which may be copyrighted.**
   - As suggested by the reviewer 1, Figure 1 was removed from the manuscript.

Response to Reviewers

Please note that the page and line numbers referred to in our responses represent those found in the newly submitted manuscript. Furthermore, specific changes are indicated in italics, where appropriate.

Reviewer 1:

1) **The reviewer suggested that information on how to identify the stink bug species was lacking and that the method for morphological identification and morphological keys should be added.**

- Lines 140-146: We included more detail about the method for morphological identification by adding, *"The two-spotted stink bug specimens were identified based on external morphological features described in literature [72] and also confirmed morphologically by an entomologist at the Agricultural Research council – Plant Health and Protection (Pretoria, South Africa). Insects used for this study are conserved in our collection located at University of Pretoria. Vouchered specimens were pinned, accession number assigned (PENT00026-PENT00030) and deposited in the National Collection of Insects located at the Agricultural Research Council – Plant Health and Protection (Pretoria, South Africa)."*

2) The reviewer suggested to submit our COI sequences to BOLD database.

- Our COI sequences were deposited to BOLD database under accession numbers PMSL01-66 sequences from Limpopo, PMSM01-45 sequences from Mpumalanga, PMSK01-46 sequences from Kwazulu-Natal. This was also stated in the revised manuscript Lines 196-199.

3) The reviewer suggested to use different names to distinguish between haplotypes.

- This is a valid suggestion. We have revised the names used for each haplotype into "*Hap_C1*" for COI, "*Hap_B1*" for Cytb and "*Hap_CB1*" for combined COI-Cytb as suggested.

4) The reviewer suggested a number of minor comments.

1. **Table 1, Table 2 and Figure 1 are presenting the same information, it seems that figure 1 is not necessary:** Figure 1 has been removed.
2. **Lines 159-160, the reviewer suggests to provide the primer names for the two cytb primers:** The two Cytb primers used in this study were developed by Muraji et al. 2000 and used in the study of Li et al. 2014. In both studies, no primer names were listed.
3. **Lines 157-172 the authors used two different PCR mastermixes:** Our initial tests of CytB amplification using a standard Taq DNA polymerase resulted in non-specific amplification. We therefore switched to using a FastStart Taq to increase the specificity. This was also added in the revised manuscript Line 176.
4. **Lines 177-181 the reviewer suggested that the details for the sequencing was not needed:** We removed the following from the manuscript *"using 5.8 µL distilled water, 1 µL Big Dye, 1 µL sequencing buffer, 0.2 µL primer and 2 µL purified PCR product. Cycle sequencing conditions included initial denaturation of 2 min at 96°C followed by 25 cycles of 10 s at 96°C, 5 s at 50°C and 4 min at 60°C"*.

5. **Line 371 *D. saccharalis* mentioned for the first time:** We are grateful to the reviewer for picking up this error. We have changed the species name to "*Diatraea saccharalis"* (line 394).

Reviewer 2:

1) **The reviewer asked to include controls in the materials and methods such as several insect outgroups (other Heteroptera) as well as several Pentatomids other than those in the same family.**
   - We included more details in line 180: *"With each run, negative and positive PCR controls were performed for PCR validation."*
   - This is a valid point, however this study was focused specifically on the genetic diversity of *Bathycoelia distincta* in South Africa and. It is our opinion that it was is not necessary to include an outgroup. For example, similar studies, published in PLoS ONE, such as Karsten et al. 2013 and Low et al. 2014 (references cited below) which were also focused on one species, did not include outgroups.

     - Karsten M, van Vuuren BJ, Barnaud A, Terblanche JS (2013) Population Genetics of *Ceratitis capitata* in South Africa: Implications for Dispersal and Pest Management. PLoS ONE 8(1): e54281. doi:10.1371/journal.pone.0054281

     - Low VL, Adler PH, Takaoka H, Ya'cob Z, Lim PE, et al. (2014) Mitochondrial DNA Markers Reveal High Genetic Diversity but Low Genetic Differentiation in the Black Fly *Simulium tani* Takaoka & Davies along an Elevational Gradient in Malaysia. PLoS ONE 9(6): e100512. doi:10.1371/journal.pone.0100512
2) **The reviewer specified that the sequencing must be replicated a minimum of three times (forward and reverse) for each individual and each primer set.**
   - Each individual was sequenced in both directions (i.e. forward and reverse primer) using an ABI Prism™ 3100 Automated Capillary DNA sequencer (Applied Biosystems). The electropherograms were visualised, base calling accuracies were checked and a consensus sequence was generated. This type of sequencing provides highly accurate results (99.99%) compared to other next generation sequencing technologies such as Illumina and/or PacBio. Sequencing each sample with each individual primer set is not compulsory in this type of study. In addition, recent studies published in PLOS ONE journal this year (references below) did not repeat the sequencing of the gene markers COI and Cytb three times. For these reasons, we did not repeat our sequencing.
   - The following sentence was added in the revised manuscript line 190: *"Electropherograms for all sequences were visualised and a consensus sequence generated"*.

   Tsoupas A, Papavasileiou S, Minoudi S, Gkagkavouzis K, Petriki O, Bobori D, et al. (2022) DNA barcoding identification of Greek freshwater fishes. PLoS ONE 17(1): e0263118. https://doi.org/10.1371/journal.pone.0263118

Arnaout Y, Djelouadji Z, Robardet E, Cappelle J, Cliquet F, Touzalin F, et al. (2022) Genetic identification of bat species for pathogen surveillance across France. PLoS ONE 17(1): e0261344. https://doi.org/10.1371/journal.pone.0261344

3) **The reviewer noted that no indication is given as to where insects tested are stored or the voucher specimens are located.**
   - The insects used in our study are conserved in our facility and some of the specimens were sent to a taxonomist at the Agricultural Research Council (ARC) in Pretoria for identification. Four of our specimens was deposited in the National Collection of Insects under the voucher numbers PENT00026-PENT00030.
   - We added additional text Lines 140-146: *"The two-spotted stink bug specimens were identified based on external morphological features described in literature [72] and also confirmed morphologically by an entomologist at the Agricultural Research Council – Plant Health and Protection (Pretoria, South Africa). Insects used for this study are conserved in our collection located at University of Pretoria. Vouchered specimens were pinned, accession number assigned (PENT00026-PENT00030) and deposited in the National Collection of Insects located at the Agricultural Research Council – Plant Health and Protection (Pretoria, South Africa)."*

Reviewer 3:

1) **The reviewer noted a few grammatical errors.**

- Line 62: we corrected "damaging pest" to "*damaging pests*"
- Line 74: we removed "," after dynamics

2) **The reviewer suggested to conduct a Mandel test to examine the association between geographic distance and genetic divergence.**

- Mantel tests were conducted and revealed no significant differences for each marker.
- The regression figures between the genetic and geographical distance among the populations for each genetic marker were added as supporting information: S1_Fig for COI, S2_Fig for Cytb and S3_Fig for COI+Cytb.
- Additional information related to these results were added in the manuscript:
  - Lines 39-40: …."*absence of correlation between genetic and geographic distance*"….
  - Lines 212-214: *"To determine the occurrence of isolation by distance (IBD), Mantel tests between the genetic and geographic distances between each population and marker were conducted using GenAlEx 6.5 with 9999 permutations [83]"*
  - Line 300: the title of the paragraph was renamed "*Genetic structure*"
  - Lines 310-313: *"Finally, for each marker, the Mantel test showed no statistically significant IBD, indicating no positive correlation between the geographic and genetic distances among B. distincta populations (COI: r = 0.731; P > 0.05; CytB:*

r = 0.310, P > 0.05; COI+Cytb: r = 0.669, P > 0.05) (S1-S3 Figs). (COI: r = 0.731; P > 0.05; CytB: r = 0.310, P > 0.05; COI+Cytb: r = 0.669, P > 0.05) (S1-S3 Figs)".

- Line 353: …*"and no IBD (P > 0.05)"….*
- Line 354: …"*S1-S3 Figs*."

3) The reviewer addressed some questions regarding line 379 - "what might cause many new mutations and haplotypes? Insecticides?" - and suggested adding some discussion to the manuscript.

- We believe that the presence of many haplotypes is linked to the species being in its native range. The species is also currently the most dominant and damaging pest of macadamia. Over time the species adapted to feed on macadamia and since nymphs are often present in orchards it suggests that macadamia orchards are a favourably habitat for reproduction and growth. Although insecticides could also likely explain some of the many haplotypes present, no information regarding insecticide resistance is currently available and we therefore did not comment on this in the manuscript
- The following sentence was added in the revised manuscript Lines 403-404: *"The high genotypic diversity suggests favourable environmental conditions for reproduction and growth of the species in its native range."*
