# Supplementary material for: Genetic diversity of the two-spotted stink bug Bathycoelia distincta (Pentatomidae) associated with macadamia orchards in South Africa
Source: PLoS One. 2022 Jun 10;17(6):e0269373. doi: 10.1371/journal.pone.0269373 (PMC9187107; doi:10.1371/journal.pone.0269373)
Supplement: Attachment — Submitted filename: Two-spotted stink bug-review comment.docx [file pone.0269373.s007.docx]

**The manuscript** entitled "Genetic diversity of the two-spotted stink bug *Bathycoelia distincta* (Pentatomidae) associated with macadamia orchards in South Africa" by Fourie et al described the genetic diversity of *B. distincta* populations in South Africa using COI and Ctyb gene markers. The population analysis using different methods have been conducted using the two markers separately and combined. It is a nice study and the manuscript provided novel information on the two-spotted stink bug. I have a couple of suggestions as below:

1) The information on how to identify the sting bug is lacking in the manuscript. The method used for the morphological identification should be added, such as which morphological keys were used and identified by whom…
2) Suggest the authors to submit the COI sequence to BOLD databases as there are no COI sequence available in the databases until now.
3) For the name of the haplotype, similar names were used for COI, cytb and COI-cytb combined. I think it will be clearer if the authors can use slightly different names to distinct them, for example, Hap_C1 for COI, Hap_B1 for cytB, Hap_CB1 for combined (Note: just my suggested name systems, do not need to follow my name conventions)? In this way, it will be easy for readers to follow when Hap_B1 mentioned, they will know it is talking about cytB haplotype.

Minor comments:

1. Table 1, Table 2 and Figure1 are presenting the same information, it seems that figure 1 is not necessary.
2. Lines 159-160: suggest providing the primer names for the two cytb primers.
3. Lines 157-172: the authors used two different PCR mastermixes to amplify the COI and cytb genes. Just wondering the reasons for the authors not to use the same matermix for amplify the two genes. According to my experience, generally it will be able to amplify the two genes under the same PCR compositions.
4. Lines 177-181: the detail pressure on how to sequence is not necessary as nowadays, it is standard protocol for the sequencing provider to run the sequence.
5. Line 371: *D. saccharalis* mentioned in the first time, the genus name should be used.

.
