# Supplementary material for: Genetic diversity of the two-spotted stink bug Bathycoelia distincta (Pentatomidae) associated with macadamia orchards in South Africa
Source: PLoS One. 2022 Jun 10;17(6):e0269373. doi: 10.1371/journal.pone.0269373 (PMC9187107; doi:10.1371/journal.pone.0269373)
Supplement: Attachment — Submitted filename: Response to Reviewers.docx [file pone.0269373.s009.docx]

Dr Patrizia Falabella

Academic Editor

PLOS ONE

**Submission of Revised Manuscript**

Thank you for your positive feedback regarding our manuscript, entitled “Genetic diversity of the two-spotted stink bug *Bathycoelia distincta* (Pentatomidae) associated with macadamia orchards in South Africa”. We believe that the suggestions made by the reviewers (both rounds) have improved the scientific quality and clarity of our manuscript. In this revision, we incorporated the suggestions made by reviewer 1, which has resulted in minor changes to our manuscript.

Please find attached our revised manuscript. Our response to the comments and suggestions made by the reviewer can be found in the document below titled “Response to Reviewers”, a marked copy of the manuscript can be found in the document titled “Revised Manuscript with Track Changes” and the unmarked version of the manuscript can be found in the document titled “Manuscript”.

We thank you in advance for your assistance in handling this manuscript further.

Kindest regards,

Dr Gerda Fourie

Corresponding author: gerda1.fourie@fabi.up.ac.za

**Journal Requirements:**

**Please review your reference list to ensure that it is complete and correct. If you have cited papers that have been retracted, please include the rationale for doing so in the manuscript text, or remove these references and replace them with relevant current references**

We have carefully reviewed our references and noted that reference [9] “DALRRD. Ressource Centre of the Department of Agriculture, Land Reform and Rural Development 2020. Available from: <https://www.dalrrd.gov.za/>.” are no longer available on-line. This reference was subsequently removed from our manuscript. The reference was not replaced since the information are also captured in reference [10].

- L56: "[9]" has been removed and as such the numbers from [9] onwards changed.

Finally, we noted a small type error in the SAMAC reference (previously numbered as [10] but [9] in the revised manuscript) and as such was updated to:

- L458-459:  " SAMAC (South African Macadamia Association). Industry statistics on the South African Macadamia Industry 2021. 2021. Available online at: <https://www.samac.org.za/industry-statistics/>"

**Response to Reviewers**

**Reviewer 1:**

**The reviewer had two minor points:**

1) **The sequence submitted in BOLD need to be publicly available before publication:**

- We made the sequences publicly available as requested. You can retrieve the sequences under the project “PBDSA-Bathycoelia distincta Pentatomidae South Africa”.

2) **Line 180 "With each PCR run, negative and positive PCR controls were performed for PCR validation"- what is the negative control? which species used, how about positive control? could the authors specify what DNA used for the controls? Was non-template control used for the PCR reactions?**

- The negative control used consisted of sample without DNA. This was to ensure that the master mix was not contaminated. This was also stated in the revised manuscript Lines 180-181. “negative controls without DNA in the PCR reaction”. We removed the mention of a positive control from the manuscript. The positive control was added to each run as an internal control. The positive control comprised of *B. distincta* DNA that previously amplified and was used as a control to ensure that all the required PCR reagents were added to the master mix correctly.
